# The Rationale and Explanation for Rehabilitation Interventions in the Management of Treatment-Induced Trismus in People with Head and Neck Cancer: A Scoping Review of Randomized Controlled Trials

**DOI:** 10.3390/medicina61081392

**Published:** 2025-07-31

**Authors:** Ernesto Anarte-Lazo, Ana Bravo-Vazquez, Carlos Bernal-Utrera, Daniel Torres-Lagares, Deborah Falla, Cleofas Rodríguez-Blanco

**Affiliations:** 1 Faculty of Health, UNIE University, 28015 Madrid, Spain; ernesto.anarte@universidadunie.com; 2 Doctoral Program in Health Sciences, University of Seville, 41009 Seville, Spain; abravovazquez@hotmail.com; 3 Physiotherapy Department, Faculty of Nursing, Physiotherapy and Podiatry, University of Seville, 41005 Seville, Spain; cbutrera@us.es (C.B.-U.); cleofas@us.es (C.R.-B.); 4 Department of Stomatology, Faculty of Dentistry, University of Seville, 41009 Seville, Spain; danieltl@us.es; 5 Centre of Precision Rehabilitation for Spinal Pain (CPR Spine), School of Sport, Exercise and Rehabilitation Sciences, University of Birmingham, Birmingham B15 2TT, UK

**Keywords:** head and neck cancer, quality of life, rehabilitation, scoping review, trismus

## Abstract

*Background and objectives*: Trismus is a frequent and debilitating complication in people with head and neck cancer (HNC) which leads to significant functional limitations and reduced quality of life. Rehabilitation interventions are commonly recommended to manage or prevent trismus. However, in many randomized controlled trials (RCTs), the theoretical justification for these interventions is poorly articulated, and the underlying biological or physiological mechanisms are not described in detail, limiting our understanding of why certain treatments may (or may not) work. This review aimed to identify and analyze how RCTs report the rationale for rehabilitation interventions and the explanations used to manage this population. *Materials and Methods*: A scoping review was conducted in accordance with the PRISMA-ScR guidelines. Five databases (PubMed, PEDro, Web of Science, Scopus, and EMBASE) were searched up to May 2025 for RCTs evaluating rehabilitation interventions for the management or prevention of treatment-induced trismus in patients with HNC. Data were extracted and synthesized narratively, focusing on the type of intervention, the rationale for its use, and the proposed mechanisms of action. *Results*: Of 2215 records identified, 24 RCTs met the inclusion criteria. Thirteen studies focused on preventive interventions—primarily exercise therapy—while the remainder addressed established trismus using exercise, manual therapy, electrotherapy, or combined treatment modalities. The rationales provided for intervention selection were heterogeneous and often lacked depth, with most studies justifying interventions based on their potential to improve mouth opening or reduce fibrosis but rarely grounding these claims in detailed pathophysiological models. Only half of the studies provided any mechanistic explanation for the intervention’s effects, and these were typically generic or speculative. *Conclusions*: RCTs investigating rehabilitation interventions for treatment-induced trismus in patients with HNC frequently lack comprehensive rationales and mechanistic explanations for their interventions. This gap limits the ability to refine and optimize treatment approaches, as the underlying processes driving clinical improvements remain poorly understood. Future research should be guided by theoretical models and include objective outcomes to better elucidate the mechanisms of action of interventions to inform clinical practice.

## 1. Introduction

Trismus, defined as mouth opening of 35 mm or less [1], is a well-known complication in people with head and neck cancer (HNC) [2,3]. While the prevalence varies enormously due to patient and physician under-reporting, it has been estimated to occur in 5–38% of patients with HNC [1,4]. It can appear early during treatment or develop progressively in the post-treatment phase, and its presence can lead to problems with speech, oral hygiene, and dental treatment, among other issues, ultimately impacting mandibular functioning and quality of life [5,6].

The etiology of trismus is multifactorial and often reflects a combination of anatomical, treatment-related, and functional mechanisms. Tumor invasion may directly infiltrate or compress the muscles of mastication, the temporomandibular joint, or the surrounding connective tissue, leading to a mechanical obstruction or even a limitation in the range of motion of the jaw due to pain [7]. In addition, in malignancies related to the oral cavity, oropharynx, nasopharynx, or infratemporal fossa, surgical resections of tumor-bearing tissues may provoke postoperative fibrosis, scarring, or motor nerve impairment [8]. However, the most frequent cause of trismus is radiation-induced fibrosis. High-dose radiotherapy, especially when applied over regions involving the masticatory apparatus, can trigger progressive fibrotic changes in muscles and soft tissues. These fibrotic changes may lead to chronic inflammation, microvascular damage, and excessive deposition of collagen, reducing tissue elasticity properties and ultimately affecting range of motion [9]. Due to increasing awareness of the functional burden of trismus, rehabilitation treatments often aim to increase maximal interincisal opening (MIO) for these patients. [10]. Nonetheless, rehabilitation approaches remain heterogenous, with limited evidence to support the effectiveness of rehabilitation interventions for trismus apart from exercise [11]. In rehabilitation, the justification/rationale for an intervention, and the proposed explanation of the mechanisms of effect are essential to ensure the translation of evidence into practice [12]. This would ensure that the understanding and development of complex interventions are aligned with the complexity of the health disorder being addressed [13]. However, in the domain of treatment-induced trismus in people with HNC, the extent to which authors report their rationale for treatment selection and the mechanisms underlying the effects of the intervention within randomized controlled trials (RCT) remains unclear.

Therefore, the aim of this scoping review is to identify and analyze how RCTs report the rationale for the chosen rehabilitation interventions used to manage or prevent treatment-induced trismus in individuals with HNC and the explanation of the mechanisms of treatment effects.

## 2. Materials and Methods

We conducted a scoping review of RCTs evaluating the management of treatment-induced trismus in people with HNC. The protocol for this review was registered in Open Science Framework (http://osf.io). Based on the framework outlined by Arksey and O’Malley [14] and later developed by Levac et al. [15], we used the PRISMA extension for Scoping Reviews (PRISMA-ScR) tool [16] to increase the quality of this study. Our review was performed based on five steps: (a) identifying the research questions, (b) searching for relevant studies, (c) selection of studies, (d) charting the data, and (e) collating, summarizing, and reporting the results.

### 2.1. Identifying the Research Question

This review aimed to address the following question: “How is the rationale for rehabilitation interventions described in RCTs for the management or prevention of treatment-induced trismus in individuals with HNC and what explanations are provided to describe the mechanisms of effect of the treatment investigated?”. Based on this question, three sub-questions were of interest:

What types of rehabilitation interventions have been evaluated in RCTs for the management or prevention of treatment-induced trismus in people with HNC?

What rationale is provided for selecting these interventions? For this purpose, the introduction section of each article was assessed.

What explanations or mechanisms of action are provided to support the expected therapeutic effects of these interventions? For this purpose, the discussion of each article was evaluated.

The participant, concept, and context (PCC) [16] framework of this scoping review can be found in Appendix A.

### 2.2. Identifying Relevant Studies

#### Eligibility Criteria

This review included RCTs involving participants with treatment-induced trismus secondary to HNC. Eligible studies had to be published in peer-reviewed journals, in English or Spanish, with publication dates up to 10 May 2025. We considered trials assessing rehabilitation interventions, including but not limited to exercise, manual therapy, electrotherapy, and other physical agents or conservative modalities. Exclusion criteria included publications in languages other than English or Spanish and studies involving surgical or pharmacological interventions.

### 2.3. Information Sources

We searched the following electronic databases: PubMed, PEDro, Web of Science, Scopus, and EMBASE. In addition, hand-searching of reference lists from relevant articles and contact with field experts were performed to identify further eligible studies.

#### 2.3.1. Search Strategy

A comprehensive search strategy was developed using a combination of Medical Subject Headings (MeSH) and free-text terms related to “Trismus,” “Rehabilitation,” and “Physical Therapy.” An example search strategy for MEDLINE is presented in Appendix B. This search strategy was designed in collaboration with a librarian and finalized through author consensus. The search was conducted by a single reviewer (EA), and the results were stored in Mendeley.

#### 2.3.2. Selecting the Studies

Following duplicate removal, two reviewers (EA and CB) independently assessed titles and abstracts according to predefined eligibility criteria and retrieved the full texts of studies meeting the eligibility criteria. Inclusion required agreement between both reviewers; disagreements were resolved through discussion or adjudicated by a third reviewer (DF) when needed. The same process was applied to the full-text screening stage by the same reviewers.

### 2.4. Charting the Data

#### 2.4.1. Data Extraction

A data extraction spreadsheet was developed, and the data were extracted by EA and checked by AB. Disagreement was resolved by a third reviewer (DF) when needed.

#### 2.4.2. Data Charting

Data extracted included authors, intervention, rationale for the application of the intervention in relation to trismus, presence or absence of positive effects of the treatment compared to a control group, and explanation of the mechanisms of effect of the treatment.

#### 2.4.3. Collating, Summarizing, and Reporting the Results

In line with the scoping review methodology, the objective of this synthesis was to map the existing literature and examine how intervention rationales and mechanisms of treatment effect are reported in RCTs addressing treatment-induced trismus. Thus, a narrative synthesis was performed, structured according to the extracted data and sub-questions. Findings were grouped thematically and presented with descriptive summaries. The full research team was involved in finalizing the synthesis and the interpretation of the results.

## 3. Results

**Figure 1:**
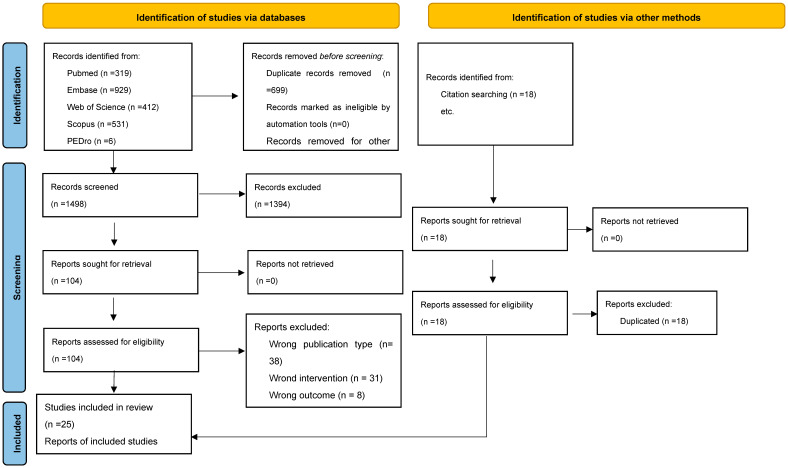
PRISMA Flow Diagram.

A total of 2215 records were identified, of which 18 were identified through hand searching and the rest through electronic database searches. After the removal of duplicates, 1498 unique records remained. Titles and abstracts were screened for eligibility, leading to the selection of 104 articles for full-text review. Of these, 25 studies met the inclusion criteria and were included in the final synthesis. The study selection process is illustrated in the PRISMA Flow Diagram (Figure 1).

A summary of the included studies is presented in Table 1.

### 3.1. Interventions

Of the 25 studies included in this scoping review, 12 focused on interventions aimed at preventing the development of treatment-induced trismus in patients undergoing therapy for HNC, with all of these prevention studies using exercise therapy as the intervention. The remaining 13 studies addressed the management of established trismus, including 2 that evaluated manual therapy alone, 2 that assessed electrotherapy alone, 5 that investigated exercise therapy as a standalone approach, and 4 that examined combined interventions such as exercise, manual therapy, and electrotherapy. In summary, 17 studies examined the effects of exercise (whether it be for prevention or management), 2 evaluated manual therapy, 2 investigated electrotherapy, and 4 assessed combination therapies.

### 3.2. Rationale

Across the 25 RCTs included in this review, we identified different rationales for the proposed interventions; thus, there was heterogeneity in both content and depth. Most studies justified the use of preventive or therapeutic active interventions based on their theoretical potential to maintain or improve the MIO, reduce fibrosis, and preserve oropharyngeal muscle function. As such, some authors cited the risk of muscle deterioration or loss of function following chemoradiotherapy [17,19], while others emphasized the potential of exercise to mitigate inflammation, endothelial injury, and radiation-induced fibrosis [20].

The rationale for manual therapy interventions was usually justified according to their capacity to relieve fascial restrictions and myofascial impairments [21]; nonetheless, some studies explicitly linked these effects to trismus-related anatomical dysfunction [41]. Studies combining multiple modalities (e.g., exercise therapy, electrotherapy, manual therapy) tended to provide more descriptive rationales but did not always clarify how each component contributed to the expected therapeutic benefit [22,25].

### 3.3. Explanation of Treatment Effects

A central finding of this review is the limited and inconsistent reporting of explanatory mechanisms that could explain the observed effects of the interventions: in 11 of 25 studies (44%), no pathophysiological explanation was provided for how the intervention might exert its therapeutic effects. In the remaining studies, explanations were often partial, generic, or speculative, with few directly linking the intervention to underlying mechanisms contributing to trismus (e.g., fibrosis, tissue remodeling, neuromuscular activation).

Nonetheless, some studies offered more integrated models. For example, it was suggested that exercise can produce an inflammatory modulation and connective tissue remodeling [32]. Moreover, vascular and thermal effects were also suggested as a potential explanation for the effects of low-level laser therapy [23]. Finally, a biomechanical connection between cervical manipulation and mandibular kinematics was also proposed as a potential explanation [40]. However, these are speculations on the potential mechanisms.

## 4. Discussion

This scoping review aimed to summarize and synthesize the existing literature describing the rationale for and explanation of treatment effects given for rehabilitation interventions evaluated in RCTs involving HNC patients with treatment-induced trismus. While many studies have assessed the effects of interventions for trismus, and some systematic reviews have been published on this topic [42,43], this is the first study to investigate why interventions are proposed and what the reasoning behind the explanation of their findings is. Our review reveals high heterogeneity in how RCTs justify and explain rehabilitation interventions for these patients. While most studies reported clinical outcomes, specifically those involving exercise therapy, these benefits were often presented without sufficient theoretical or mechanistic context.

Overall, these findings reflect positive trends for rehabilitative interventions—especially those combining exercise and manual therapy—but also highlight significant variability across trials. Most of the studies were based on active interventions, demonstrating a trend to involve the patient in their treatment. However, despite the overall recognition of the need for early or preventive interventions, only a few studies grounded their rationale in pathophysiological models specific to trismus or to the sequelae of cancer treatment. Among them, the most frequent explanation was a reduction in treatment-induced fibrosis after the intervention [20,21,31,33,40]. Other explanations were a reduction in inflammation [32,35] or an increase in vascularization [24,34,38,40]. However, 11 studies of 25 did not include a pathophysiological explanation for their findings. This lack of explanation may limit the replicability, refinement, and clinical translation of the interventions evaluated.

In contrast to the observations in this scoping review, some evidence has suggested the rationale for and/or explanation of the mechanisms involved in the effectiveness of rehabilitation treatments in temporomandibular disorders. For example, it has been suggested that manual therapy can improve mouth opening as a result of the restoration of joint glide, reduction in capsular rigidity, and analgesic effects mediated by the central nervous system and peripheral mechanisms [44]. Moreover, exercise therapy of the craniomandibular muscles may improve mouth opening by increasing blood perfusion, reducing muscle fatigue, and promoting neuromuscular plasticity [45]. In the field of treatment-induced trismus, there has been enormous progress in rehabilitation research, with the proposal and recognition of different treatments for the management of treatment-induced trismus in people with HNC. However, we cannot currently provide an evidence-based explanation for how or why even exercise, the most studied intervention, produced changes. Understanding of how and why rehabilitation is offered to patients is an essential starting point towards more responsive rehabilitation services [46].

In health research, connecting the design of interventions and the selection of outcomes with the understanding of the mechanisms of effect on an intervention is essential [47]. Without a clear understanding of the physiological mechanisms exerted by a treatment, we would be unable to identify which components of a treatment are truly beneficial, which are redundant, or how we could optimize future RCT protocols [48]. It has been recognized that the effectiveness of a rehabilitative intervention will be influenced by many uncontrollable and interrelated factors [49]. It is notable that, with regard to manual therapy, biomechanic and/or pathological explanations were more frequently suggested, indicating a likely improvement in myofascial structures [21] or an improvement via the close biomechanical relationship between the temporomandibular joint and cervical spine [41]. However, this is not in line with previously published models of manual therapy, which suggested that the effects of this kind of treatment should not only be considered in anatomical and/or biomechanical terms but also from a neurophysiological point of view [50]. Thus, it would be interesting to investigate the mechanisms underlying the effects of manual therapy for trismus, especially considering that different types of manual therapy interventions can be applied and could achieve their effect via different mechanisms [50]. In that sense, the UK Medical Research Council (MRC) framework for complex interventions highlights the need to ground intervention development in both a theoretical and empirical understanding, including a clear explanation of the causal mechanisms linking an intervention component to outcomes [51]. Thus, rehabilitation interventions should be grounded in a mechanistic model that explains how the treatment components are expected to produce change, considering factors such as neuromuscular plasticity, facial dynamics, or tissue remodeling.

### 4.1. Study Limitations

One of the main limitations of this scoping review is that we only included RCTs; other studies, such as prospective studies, may include other rationales/and or explanations that could help us to understand the mechanisms proposed for the different treatments more deeply. Moreover, the heterogeneity in protocols and the lack of standardization in reporting made it difficult to compare different studies and to reach firm conclusions. In addition, in line with the purpose and methodology of a scoping review, we did not perform a formal risk-of-bias or quality assessment of the included RCTs, as our goal was to map the available evidence rather than critically evaluate it. Additionally, we restricted the inclusion criteria to studies published in English or Spanish, which may have introduced a language-based selection bias. It is possible that relevant studies published in other languages were missed, potentially limiting the comprehensiveness of our findings. Finally, the rationale and/or explanations were limited, with no studies performing a real assessment of the mechanisms produced by their intervention, which would be necessary to validate proposed mechanisms. This represents a significant gap in the literature and limits the replication and advancement of mechanistically informed rehabilitation approaches.

### 4.2. Implications for Future Research

Despite many studies in this field, very few studies employed treatment theory or mechanistic frameworks to articulate how and why a specific intervention was expected to improve trismus outcomes. This observation indicates a need for a stronger theoretical underpinning in future trials. Understanding these mechanisms could facilitate the reproducibility of studies and enhance study design. In that sense, comprehension of the mechanisms of effect of different interventions may guide future trials to combine different therapies to maximize treatment benefit. Moreover, future studies should consider the use of assessment techniques that allow for objective measures of the changes that occur at a pathoanatomical and/or physiological level to improve our understanding of the mechanisms underlying post-treatment changes. For example, future studies could consider evaluating masticatory muscles with ultrasound or electromyography, before and after the intervention.

## 5. Conclusions

This scoping review summarizes the literature describing the rationale for a chosen treatment and the explanation of treatment effects of interventions for the management and prevention of trismus in people with HNC. The most common rationale for and explanation of therapeutic effects provided was the reduction in treatment-induced fibrosis. Nonetheless, there is a general lack of knowledge about the proposed mechanisms underlying the therapeutic effects of the studied interventions. Standardization of protocols and outcome measurements, as well as the inclusion of diverse methodological designs that complement the evidence generated by RCTs, is needed.

## Figures and Tables

**Table 1:** Summary of the included studies.

Study	Intervention	Rationale	Findings	Explanation
Hajdú et al., 2022 [17]	Preventive exercise therapy	Loss of body mass in HNC and loss of functionality in swallowing	At the end of the treatment, significant effects on mouth opening were found (*p* = 0.01)	Absence of explanation of pathophysiological mechanisms
Petersson et al., 2024 [18]	Preventive exercise therapy	Exercise is effective but adherence is low: there is a need for simple protocols	At follow-up, no significant effects on mouth opening were found (0.78)	Absence of explanation of pathophysiological mechanisms
Messing et al., 2017 [19]	Preventive exercise therapy	Swallowing exercises lead to less muscle deterioration	Significant differences were found 24 months after CRT (*p* = 0.04)	Absence of explanation of pathophysiological mechanisms
Saghafi et al., 2024 [20]	Preventive exercise therapy	Exercise can improve radiation-induced inflammation, hypoxia, endothelial injury, and fibrosis	At 6 and 12 months from baseline, significant differences were found (*p* = 0.01 and *p* = 0.012, respectively).	Jaw exercises may help to improve subcutaneous fibrosis
Ortiz-Comino et al., 2022 [21]	Manual therapy	Manual therapy can relieve fibrosis and fascial restrictions	After treatment, MIO was significantly higher in the IG	Manual therapy may improve myofascial impairments. The fact of including intra- and extraoral techniques allowed the researchers to cover all the musculature potentially involved in trismus
Dai et al., 2024 [22]	Exercise therapy + manual therapy	Exercise can reverse damage to temporomandibular structures	3 months after radiotherapy, the IG demonstrated statistically significant differences (*p* = 0.001)	Absence of explanation of pathophysiological mechanisms
Hu et al., 2021 [23]	Exercise therapy	Resistance exercise improves perception of fatigue and reduces symptom severity	12 weeks post-discharge, the IG demonstrated less difficulties in mouth opening (*p* = 0.049)	Absence of explanation of pathophysiological mechanisms
Elgohary et al., 2018 [24]	Electrotherapy + exercise therapy	LIUS: the increase in local tissue temperature and improvement in flexibility and extensibility of tissue may improve trismus LLLT: the reduction in swelling and subsequent pain may help in trismus TET: may help to improve functional movements and diminish pain	Both interventions demonstrated significant differences when compared to the control group (*p* < 0.001)	LIUS can reduce inflammation, improve blood circulation to the target tissue, produce micro massage, remove waste products, accelerate lymphatic drainage, improve metabolic activities, and increase the extensibility of soft tissue. Absence of explanation of the pathophysiological mechanisms of LLLT
Wang et al., 2019 [25]	Exercise therapy + manual therapy + thermotherapy	Thermotherapy: reduces pain and relieves muscle tension Manual therapy: improves pain Exercise: improves trismus	Twelve weeks after baseline, the IG demonstrated higher MIO that the CG (*p* < 0.001)	Absence of explanation of pathophysiological mechanisms
Lee et al., 2018 [26]	Electrotherapy	Pain relief can help to increase mouth opening	No statistical differences were found	Since no changes were found in pain or function, and TENS aims to improve function through a reduction in pain, function did not improve
Wen et al., 2022 [27]	Exercise therapy	Exercise can help to improve negative effects of cancer treatment	At the 12-week follow-up, the improvement in mouth opening difficulties was higher in the IG (*p* = 0.009)	Exercise can reverse the diverse effects of radiation, such as inflammation, cell death, and matrix remodeling
Hogdal et al., 2015 [28]	Preventive exercise therapy	Positive effect on swallowing and maximal interincisor distance	The early exercise intervention showed no significant benefits in MID at 12 months (*p* = 0.07)	Absence of explanation of pathophysiological mechanisms
Carnaby-Mann et al., 2012 [29]	Preventive exercise therapy	Swallowing exercises facilitate maintenance of oropharyngeal muscle function	The functional swallowing ability deteriorated less (*p* ≤ 0.03) in the IG than in the CG or SG (*p* < 0.06)	The reduction in relaxation time and maintenance of muscle size might reflect a deterrent to inflammatory changes. The reduction in muscle edema or fatty infiltration is likely to be a contributing factor. Absence of explanation of pathophysiological mechanisms
Bragante et al., 2020 [30]	Preventive exercise therapy	Preventing the development of trismus	There was no significant difference in MO measure between the groups at the 3 assessment time points (*p* = 0.264)	Absence of explanation of pathophysiological mechanisms
Sekar et al., 2024 [31]	Exercise therapy and manual therapy	Exercise therapy is useful for increasing jaw range of motion and reducing pain. Myofascial release of the restricted fascia around the head and neck may help alleviate facial and neck pain	The IG demonstrated statistically significant differences (*p* = 0.002)	Therapeutic massage increases blood flow and relaxes the masticatory muscles, as do exercises to break down myofascial adhesions and fibrosis, especially in the first six weeks of treatment
Loorents et al., 2014 [32]	Preventive exercise therapy	Device developed specifically for trismus	There were no significant differences in MIO between the IG and CG	Absence of explanation of pathophysiological mechanisms
Yang et al., 2025 [33]	Preventive exercise therapy	Early exercises are particularly effective in preventing trismus	The MIO in the experimental group was significantly higher than that in the control group. This difference persisted in the twelfth week post-surgery (three weeks after the end of radiotherapy) (38.778 ± 1.267 mm vs. 35.167 ± 1.254 mm, *t* = 12.154, *p* < 0.001)	Regular mouth opening activities can inhibit excessive scar tissue formation to some extent, helping to maintain or enhance mouth opening. Early mouth opening exercises can reduce the risk of inflammation and fibrosis by increasing local blood circulation and tissue elasticity before radiation damage occurs, thus protecting mouth opening function to some extent
Fong et al., 2015 [34]	Exercise therapy	Short-term Qigong training can reduce neck–shoulder pain and disability in individuals with neck pain	The deterioration in mouth opening capacity appears to have been less severe in the Tai Chi Qigong group (*p* = 0.181) than in the control group (*p* < 0.001) over time	Possible mechanisms include range-of-motion exercises increasing temporomandibular joint mobility and improving the flexibility and elasticity of the connective tissues surrounding the joint and Tai Chi Qigong training improving blood circulation and decreasing the local inflammatory response
Retèl et al., 2016 [35]	Preventive exercise therapy	TheraBite has proven its effectiveness in both preventive and treatment settings for trismus	After treatment, trismus was significantly lower in the IG	Absence of explanation of pathophysiological mechanisms
Van der Molen et al., 2011 [36]	Preventive exercise therapy	Tolerance of TheraBite is good, it is easy to use, and compliance tends to be high	Comparing the pre- and post-treatment maximum mouth opening (MIO), a significant decrease over time was found (from 50 to 47 mm, respectively; *p* < 0.01), but not in the occurrence of trismus (MIO < 35 mm; from 5 to 7 patients; *p* = 0.70)	Training seems to have a positive effect on the presence of post-swallow residue after concomitant chemoradiotherapy, which might suggest better mucosal clearance and improved muscle activity. Absence of explanation of pathophysiological mechanisms
Lee et al., 2018 [37]	Preventive exercise therapy	Relieve or prevent trismus	The difference between the two interventions was not significant, although the power of the study was low, because the authors failed to achieve the target recruitment rate and the attrition rate was higher than anticipated. The estimated difference in mean mouth opening at six months after was −2.43 mm. This was not significant (*p* = 0.39)	Absence of explanation of pathophysiological mechanisms
Aboelez et al., 2025 [38]	Electrotherapy	Combined trismus appliances improve mouth opening. Threaded tapered screw appliance gradually wedges the teeth apart. Low-level laser therapy helps the tissue heal by reducing pain and swelling and reducing inflammatory conditions	There was a statistically significant difference at different times of evaluation within all groups (*p* < 0.0001). Group C recorded the lowest values for trismus symptoms, followed by group B, followed by group A. For example, the three groups recorded values of (9.83 ± 1.75), (18.00 ± 2.09), (21.33 ± 2.02), respectively, in the item describing problems yawning in the last six months of the evaluation period	Both therapies could be explained by the fact that both increase vasodilation
Sirapracha et Sessirisombat, 2018 [39]	Exercise therapy	The most convenient and widely used technique to improve trismus is jaw exercise	At 1 year after radiotherapy, the average percentage of MIO reduction in the dynamic group was greater compared with the static group; however, the difference was not significant (*p* = 0.706)	It is possible that jaw exercise with the tongue blade stack (static group) stretched the masticatory muscles continuously and the force was greater compared with the force from the patients’ fingers (dynamic group). The force from the patients’ fingers was related to the patients’ physical strength and their ability to separate their fingers
Tang et al., 2011 [40]	Exercise therapy	Effective therapeutic effect	The mean interincisor distance in patients of both groups decreased at the 3-month follow-up; the decrease in the rehabilitation group was less than that of the control group (0.19 ± 0.5 cm vs. 0.69 ± 0.56 cm, *p* = 0.004)	Range-of-motion exercises for the jaw and tongue and resistance exercises could strengthen the musculature, increase mobility, and improve the flexibility and elasticity of the temporomandibular joint. In addition, exercises may improve blood circulation
Castro-Martín et al., 2021 [41]	Manual therapy	MIT can assist in eliminating functional limitations by helping to restore general health and reducing or eliminating pain owing to its systemic effects	MIO improved significantly after MIT (score change + 3.36 mm; *p* < 0.004) but not after the placebo session (score change −0.36).	One session of MIT on the cervical region increased MIO; this may be explained by the dynamic biomechanical relationship that exists between the cervical spine and the temporomandibular joint during active mouth opening

CG: control group; F-UP: follow-up; IG: intervention group; LIUS: low-intensity ultrasound; LLLT: low-level laser therapy; MIO: maximal interincisal opening; MIT: myofascial induction therapy; TET: tradition exercise therapy; SG: sham group; TENS: Transcutaneous Electrical Nerve Stimulation.

## Appendix A

**Table A1:** The participant, context, and concept framework used in this study for defining the eligibility of the studies for the primary research question.

P: Population	People with treatment-induced trismus associated with head and neck cancer
C: Concept	Characteristics of different rehabilitation modalities for the management of treatment-induced trismus in people with head and neck cancer
C: Context	Studies including observational or interventional trials are of interest for this review if they include the implementation of rehabilitation strategies for the management of trismus

## Appendix B

**Table A2:** Search strategy on Pubmed.

Search Strategy	
	Terms
#1	“Physical Therapy Modalities” “[Mesh]”
#2	“Rehabilitation” “[Mesh]”
#3	“Education” “[Mesh]”
#4	“Exercise” “[Mesh]”
#5	“Exercise Therapy” [Mesh]
#6	“Electrotherapy” “[Mesh]”
#7	“Manual Therapy” “[Mesh]”
#8	“jaw exercises”
#9	“physiotherapy”
#10	“physical therapy”
#11	“Trismus” “[Mesh]”
#12	“trismus”
#13	“mouth opening”
#14	“jaw opening”
#15	“Neck” “[Mesh]”
#16	“Head” “[Mesh]”
#17	“Neoplasms” “[Mesh]”
#18	“Head and Neck Neoplasm” “[Mesh]”
#19	“head and neck cancer”
#20	#1 OR #2 OR #3 OR #4 OR #5 OR#6 OR #7 OR #8 OR #9 OR #10
#21	#11 OR #12 OR #13 OR #14
#22	#15 OR #16 OR #17 OR #18 OR #19
#23	#20 AND #21 AND #22
